# Study of three interesting *Amanita* species from Thailand: Morphology, multiple-gene phylogeny and toxin analysis

**DOI:** 10.1371/journal.pone.0182131

**Published:** 2017-08-02

**Authors:** Benjarong Thongbai, Steven L. Miller, Marc Stadler, Kathrin Wittstein, Kevin D. Hyde, Saisamorn Lumyong, Olivier Raspé

**Affiliations:** 1 Centre of Excellence in Fungal Research, and School of Science, Mae Fah Luang University, Chiang Rai, Thailand; 2 Botany Department, University of Wyoming, Laramie, Wyoming, United States of America; 3 Department of Microbial Drugs, Helmholtz Centre for Infection Research; and German Centre for Infection Research (DZIF), partner site Hannover/Braunschweig, Braunschweig, Germany; 4 Department of Biology, Faculty of Science, Chiang Mai University, Chiang Mai, Thailand; 5 Botanic Garden Meise, Meise, Belgium; 6 Fédération Wallonie–Bruxelles, Service général de l'Enseignement universitaire et de la Recherche scientifique, Bruxelles, Belgium; Universita degli Studi di Napoli Federico II, ITALY

## Abstract

*Amanita ballerina* and *A*. *brunneitoxicaria* spp. nov. are introduced from Thailand. *Amanita fuligineoides* is also reported for the first time from Thailand, increasing the known distribution of this taxon. Together, those findings support our view that many taxa are yet to be discovered in the region. While both morphological characters and a multiple-gene phylogeny clearly place *A*. *brunneitoxicaria* and *A*. *fuligineoides* in sect. *Phalloideae* (Fr.) Quél., the placement of *A*. *ballerina* is problematic. On the one hand, the morphology of *A*. *ballerina* shows clear affinities with stirps *Limbatula* of sect. *Lepidella*. On the other hand, in a multiple-gene phylogeny including taxa of all sections in subg. *Lepidella*, *A*. *ballerina* and two other species, including *A*. *zangii*, form a well-supported clade sister to the *Phalloideae* sensu Bas 1969, which include the lethal “death caps” and “destroying angels”. Together, the *A*. *ballerina*-*A*. *zangii* clade and the Phalloideae sensu Bas 1969 also form a well-supported clade. We therefore screened for two of the most notorious toxins by HPLC-MS analysis of methanolic extracts from the basidiomata. Interestingly, neither α-amanitin nor phalloidin was found in *A*. *ballerina*, whereas *Amanita fuligineoides* was confirmed to contain both α-amanitin and phalloidin, and *A*. *brunneitoxicaria* contained only α-amanitin. Together with unique morphological characteristics, the position in the phylogeny indicates that *A*. *ballerina* is either an important link in the evolution of the deadly *Amanita* sect. *Phalloideae* species, or a member of a new section also including *A*. *zangii*.

## Introduction

*Amanita* sect. *Phalloideae* (Fr.) Quél., is characterized by a non-appendiculate and non-striate pileus, a persistent partial veil, and a limbate or saccate volva on the bulbous stipe base [1–4]. The diagnostic microscopic features include amyloid basidiospores and a pileipellis composed of gelatinous filamentous hyphae [1–4]. The type species of this section is the “Euro-Asian Death Cap”, *A*. *phalloides* (Fr.) Link., first described from Europe [5]. The sect. *Phalloideae* currently comprises approximately 61 taxa that have been described worldwide [6,7]. The cyclooligopeptide toxins found in members of sect. *Phalloida*e, which include amatoxins, phallotoxins, and virotoxins, have been studied for over a decade [8–10]. The amatoxins, including α-amanitin and β-amanitin, are considered ten times more deadly than phallotoxins [11].

Members of sect. *Phalloideae* are consumed after mistaking them for edible species. In Asia, confusion most often occurs with other *Amanita* spp. in sect. *Caesareae* Singer ex Singer, which contains a number of edible species [12]. Recently, a multiple gene phylogenetic analysis rigorously examined species in sect. *Phalloideae* [13, 14] and sect. *Caesareae* [15], with the aim of exploring biogeographic history and plesiomorphies that may help to resolve evolutionary relationships among deeper clades of these two sections. In order to understand the evolutionary relationships in sect. *Phalloideae*, researchers have begun to critically evaluate the toxins, toxin encoding genes, and biogeography to support the taxonomy [11, 13, 14, 15].

In a recent publication resulting from biodiversity studies in northern Thailand we documented two first records of sect. *Phalloideae*, *A*. *rimosa* P. Zhang & Zhu L. Yang with white basidiomata and *A*. *zangii* Zhu L. Yang, T.H. Li & X.L. Wu [16], although the placement of the latter in sect. *Phalloideae* is controversial because of very atypical morphology [13, 16]. In the present paper, we report two additional species in *Amanita* sect. *Phalloideae*, and a third species whose taxonomic placement is also problematic, with two of the species new to science The macro- and micro-morphology of those three taxa are described, and photographs and line drawings are provided. Moreover, their phylogenetic affinities are discussed based on multigene phylogenetic analyses (rpb2, β-tubulin, nrITS, and nr5.8S). Finally, we report on the screening of the three species for toxins known to occur in *Amanita*, including α-amanitin and phalloidin.

## Materials and methods

### Collections

Specimens were collected mainly in forests dominated by *Fagaceae* (*Castanopsis*, *Lithocarpus*, *Quercus*) and/or *Dipterocarpaceae* (*Dipterocarpus*, *Shorea*) during the rainy season. The rainy season in the north (including Chiang Mai and Chiang Rai provinces) normally occurs from May to beginning of October, whereas the rainy season in the south (including Song Khla Province) occurs from May to December. Fresh specimens were photographed and described, and then dried using a food dehydrator (at ca. 40–50°C). Tissue samples were taken from fresh basidiomata with aseptic technique and kept in 10% CTAB (cetyl trimethylammonium bromide) for later DNA analyses. The examined specimens were deposited in either one or two of the following herbaria: Mae Fah Luang University, Thailand (MFLU), Botanic Garden Meise, Belgium (BR), and Chiang Mai University, Thailand (SDBR-CMU). Herbarium codes follow Index Herbariorum [17], with the exception of "RET", which is the code adopted for R.E. Tulloss' Herbarium Rooseveltensis Amanitorum, and HKAS, which stands for the Cryptogamic Herbarium of Kunming Institute of Botany. All author citations of species rank not included in the main body of the text are located in **Table 1**.

**Table 1:** Taxa of *Amanita* included in molecular phylogenetic analysis. Newly generated sequences in this study are highlighted in bold.

Species	Voucher	Country	GenBank accession no.				Reference
			ITS	LSU	rpb2	β-tubulin	
**Subgenus *Lepidella***							
*Incertae sedis*							
***Amanita ballerina* sp. nov.**	**OR1014**	**Thailand**	**KY747466**	–	**KY656883**	**KY656864**	
***Amanita ballerina* sp. nov.**	**OR1026**	**Thailand**	**KY747467**	–	**KY656884**	**KY656865**	
*Amanita zangii*	GDGM29241	China	KJ466432	KJ466499	KJ466668	KJ466588	[13]
***Amanita zangii***	**OR1220**	**Thailand**	**KY747470**	–	**KY656887**	**KY656868**	
***Amanita zangii***	**OR1224**	**Thailand**	**KY747472**	–	**KY656889**	**KY656870**	
*Amanita* sp.	HKAS77321	China	KJ466416	KJ466481	KJ466646	KJ466560	[13]
Sect. *Phalloideae*							
*Amanita amerivirosa* nom. prov.	RET 480–1	USA	KJ466399	KJ466461	KJ466630	KJ466544	[13]
***Amanita brunneitoxicaria* sp. nov.**	**BZ2015-01**	**Thailand**	**KY747462**	–	**KY656879**	**KY656860**	
***Amanita brunneitoxicaria* sp. nov.**	**BZ2015-02**	**Thailand**	**KY747463**	–	**KY656880**	**KY656861**	
*Amanita exitialis* Zhu L. Yang & T.H. Li	HKAS74673	China	KJ466375	KJ466435	KJ466590	KJ466502	[13]
*Amanita exitialis*	HKAS75774	China	JX998027	JX998052	KJ466591	KJ466503	[38]
*Amanita fuliginea* Hongo	HKAS77132	China	KJ466376	KJ466436	KJ466598	KJ466510	[13]
*Amanita fuliginea*	HKAS79685	China	KJ466377	KJ466437	KJ466594	KJ466506	[13]
*Amanita fuliginea*	HKAS77343	China	KJ466401	KJ466464	KJ466633	KJ466547	[6]
*Amanita fuligineoides*	HKAS52727	China	JX998024	JX998047	KJ466599	KJ466511	[38]
*Amanita fuligineoides*	LHJ140722-13	China	KP691685	KP691696	KP691705	KP691715	[39]
***Amanita fuligineoides***	**BZ2013-41**	**Thailand**	**KY747460**	–	**KY656877**	**KY656858**	
***Amanita fuligineoides***	**OR1044**	**Thailand**	**KY747468**	–	**KY656885**	**KY656866**	
*Amanita griseorosea* Qing Cai, Zhu L. Yang & Y.Y. Cui	HKAS77334	China	KJ466413	KJ466476	KJ466661	KJ466580	[6]
*Amanita molliuscula* Qing Cai, Zhu L. Yang & Y.Y. Cui	HKAS75555	China	KJ466408	KJ466471	KJ466638	KJ466552	[6]
*Amanita molliuscula*	HKAS77324	China	KJ466409	KJ466472	KJ466639	KJ466553	[6]
*Amanita ocreata* Peck	HKAS79686	USA	KJ466381	KJ466442	KJ466607	KJ466518	[13]
*Amanita pallidorosea* P. Zhang & Zhu L. Yang	HKAS77327	China	KJ466386	KJ466446	KJ466608	KJ466519	[13]
*Amanita pallidorosea*	HKAS61937	China	KJ466382	KJ466443	KJ466609	KJ466520	[13]
*Amanita phalloides* Secr.	HKAS75773	USA	JX998031	JX998060	KJ466612	KJ466523	[38]
*Amanita rimosa*	HKAS75777	China	JX998018	JX998044	KJ466615	KJ466526	[38]
*Amanita sturgeonii* nom. prov.	RET 422–8	USA	KJ466406	KJ466469	KJ466649	KJ466563	[13]
*Amanita sturgeonii* nom. prov.	RET 493–6	USA	KJ466407	KJ466470	KJ466650	KJ466564	[13]
*Amanita suballiacea*	RET 478–6	USA	KJ466419	KJ466484	KJ466600	KJ466512	[6]
*Amanita suballiacea* P. Zhang & Zhu L. Yang	RET 490–1	USA	KJ466420	KJ466485	KJ466601	KJ466513	[6]
*Amanita subfuliginea* Qing Cai, Zhu L. Yang & Y.Y. Cui	HKAS77326	China	KJ466404	KJ466467	KJ466636	KJ466550	[6]
*Amanita subfuliginea*	HKAS77347	China	KJ466405	KJ466468	KJ466637	KJ466551	[6]
*Amanita subjunquillea* S. Imai	HKAS63418	China	KJ466423	KJ466488	KJ466651	KJ466569	[6]
*Amanita subjunquillea*	HKAS74993	China	KJ466424	KJ466489	KJ466652	KJ466570	[6]
*Amanita subpallidorosea* Hai J. Li	HKAS77350	China	KJ466400	KJ466462	KJ466631	KJ466545	[39]
*Amanita subpallidorosea*	LHJ140922-32	China	KP691679	KP691689	KP691698	KP691709	[39]
*Amanita subpallidorosea*	LHJ140923-02	China	KP691676	KP691690	KP691699	KP691710	[39]
*Amanita virosa* Bertill.	HKAS56694	China	JX998030	JX998058	KJ466664	KJ466583	[38]
*Amanita virosa*	HMJAU23304	China	KJ466431	KJ466498	KJ466667	KJ466587	[38]
*Amanita* sp. 8	HKAS75150	Bangladesh	KJ466414	KJ466477	KJ466641	KJ466555	[13]
*Amanita* sp. 9	HKAS77323	China	KJ466415	KJ466478	KJ466642	KJ466556	[13]
*Amanita* sp. 10	HKAS77322	Australia	KJ466395	KJ466457	KJ466643	KJ466557	[13]
Sect. *Lepidella*							
***Amanita atrobrunnea*** Thongbai, Raspé & K.D. Hyde	**BZ-N09**	**Thailand**	**KY747455**	KT934314*	**KY656871**	**KY656852**	* [36]
***Amanita macrocarpa*** W.Q. Deng, T.H. Li & Zhu L. Yang	**OR1223**	**Thailand**	**KY747471**	–	**KY656888**	**KY656869**	
***Amanita* cf. *manginiana***	**BZ-N11**	**Thailand**	**KY747457**	**KY747474**	**KY656873**	**KY656854**	
*Amanita modesta* Corner & Bas	HKAS75405	China	KJ466379	KJ466439	KJ466605	KJ466517	[13]
*Amanita modesta*	HKAS79688	China	**–**	KJ466440	KJ466604	KJ466516	[13]
***Amanita* cf. *oberwinklerana***	**BZ2013-39**	**Thailand**	**KY747459**	**KY747476**	**KY656876**	**KY656857**	
*Amanita pseudoporphyria* Hongo	HKAS56984	China	KC429050	KJ466450	KJ466614	KJ466525	[13]
***Amanita pseudoporphyria***	**BZ-N10**	**Thailand**	**KY747456**	**KY747473**	**KY656872**	**KY656853**	
*Amanita vestita* Corner & Bas	HKAS79687		**–**	KJ466494	KJ466662	KJ466581	[13]
*Amanita virgineoides* Bas	HKAS79691	China	**–**	KJ466495	KJ466663	KJ466582	[13]
Sect. *Validae*							
*Amanita* aff. *fritillaria*	HKAS56832	China	KJ466372	KJ466479	KJ466644	KJ466558	[13]
***Amanita* cf. *spissacea***	**BZ2015-40**	**Thailand**	**KY747464**	–	**KY656881**	**KY656862**	
***Amanita* cf. *spissacea***	**OR1214**	**Thailand**	**KY747469**	**KY747478**	**KY656886**	**KY656867**	
***Amanita* sp.**	**BZ2013-71**	**Thailand**	**KY747461**	–	**KY656878**	**KY656859**	
Sect. *Amidella*							
*Amanita* sp.	HKAS77339	China	KJ466417	KJ466482	KJ466647	KJ466561	[13]
*Amanita* sp.	HKAS77340	China	KJ466418	KJ466483	KJ466648	KJ466562	[13]
**Subgenus *Amanita***							
Sect. *Amanita*							
***Amanita concentrica* T. Oda, C. Tanaka & Tsuda**	**BZ2013-26**	**Thailand**	KU904816*	KU877534*	**KY656875**	** KY656856**	*[16]
***Amanita rubrovolvata* S. Imai**	**BZ2015-68**	**Thailand**	**KY747465**	**KY747477**	**KY656882**	**KY656863**	
***Amanita* cf. *sinensis***	**BZ-N17**	**Thailand**	**KY747458**	**KY747475**	**KY656874**	**KY656855**	
*Amanita subfrostiana* Zhu L. Yang	HKAS57042	China	JN943173	JN941162	JQ031118	KJ466565	[13]
*Amanita subglobosa* Zhu L. Yang	HKAS58837	China	KU248106	AF024478	KJ466567	JQ031121	[13]

### Morphological study

Macro-morphology was described from fresh specimens. Color codes are according to Kornerup & Wanscher (1978). The colour change reaction with 10% KOH was tested on the stipe surface of *A*. *ballerina*, as is done in other species with white basidiomata [18]. Microscopic features were studied from dried tissue mounted in H_2_O and 5% KOH aqueous solution. Congo red was used for highlighting all tissues, and amyloidity of basidiospores was observed using Melzer reagent. Dimensions of microscopic structures were measured using Image Frame Work (Tarosoft®, Thailand). Basidiospore measurements are accompanied with the following notation: "[*n*/*m*/*p*]", which indicates that *n* basidiospores were measured from *m* basidiomata of *p* collections, with a minimum of 25–50 basidiospores from each basidiome. Size and shape of basidiospores are presented in a form following the description of ranges for biometric variables according to Tulloss [18] (*a*–) *b*–*c* (–*d*), in which *b* represents the 5^th^ percentile, *c*, the 95^th^ percentile, while *a* and *d* are the lowest and highest extreme values measured, respectively. The average length is indicated as **L'** whereas **W'** is the average width. The range of length/width ratio of basidiospores (Q) is provided. In addition to Tulloss' standard format, standard deviationis provided for **Q'** (the mean of all Q values computed for a single taxon). Faces of Fungi [19], Index Fungorum [20] and MycoBank [21] numbers are provided.

### DNA isolation, amplification and sequencing

Specimens were processed for molecular analyses at two core facilities including the Botany Department, University of Wyoming (UW), USA and Botanic Garden Meise, Belgium, using a variety of methodologies for extraction of genomic DNA, PCR and sequencing. Genomic DNA extractions at the University of Wyoming were performed using a CTAB protocol with phenol-chloroform-isoamyl alcohol purification, followed by cleaning with a silica-matrix binding procedure [22]. At Botanic Garden Meise, DNA extractions were performed using a slightly different CTAB protocol. PCR amplification of ITS (nuclear ribosomal internal transcribed spacer) and LSU (large subunit ribosomal DNA) was performed using the primer pairs ITS4/ITS5 or ITS1-F/ITS4, and LR0R/LR5, respectively. Parts of the protein-coding genes β-tubulin and rpb2rpb2 (second largest subunit of RNA polymerase II) were amplified using the primer pairs Am-β-tub-F/Am-β-tub-R and Am-6F/Am-7R, respectively [13]. Purified PCR products were then sequenced at the Nucleic Acid Exploration Facility at the University of Wyoming on an ABI 3130 XL DNA analyzer (Applied Biosystems), or at Macrogen Europe (Amsterdam) on an ABI 3730 XL DNA analyzer (Applied Biosystems), using the same primer combinations as for PCR, except for Am-β-tub-F, which was replaced by the shorter primer Am-β-tub-F-Seq (5’-CGGAGCRGGTAACAAYTG-3’). Forward and reverse reads were assembled and edited with Geneious Pro 5.1.7 (Biomatters Ltd., Auckland, New Zealand).

### DNA sequence dataset assembly

Seventy-six sequences of collections from Thailand were newly generated for this study and deposited in GenBank (GB) (http://www.ncbi.nlm.nih.gov/; Table 1). Initial BLAST searches (http://blast.ncbi.nlm.nih.gov) of both LSU and ITS1+5.8S+ITS2 sequences were performed to estimate similarity with *Amanita* sequences already in GB. Additional sequences were identified using phylogenetic inferences and were also retrieved from GB (**Table 1**). The quality of the sequences was considered in selecting sequences from GB for use in the analyses. Because some of the species studied here, namely *Amanita ballerina* sp. nov. and *A*. *zangii*, have morphological characters atypical for sect. *Phalloideae* and/or have been successively placed in different sections in the past, the taxon sampling was performed to cover not only section *Phalloideae*, but also the three other sections in subgen. *Lepidella*, namely sect. *Lepidella*, sect. *Validae*, and sect. *Amidella*, as well as five taxa from subgen. *Amanita*, sect. *Amanita* selected as outgroup. Because LSU is of restricted phylogenetic utility (see e.g., Tulloss [23] for a brief discussion), it was not included in the phylogenetic analyses.

### Phylogenetic analyses

Sequences were initially aligned with MAFFT v.7.0 [24] using the G-INS-i iterative refinement algorithm, with minimal manual adjustment in BioEdit v.7.0.9 (Hall 1999). Introns of protein-coding genes were excluded from the analyses, mainly because they were not included in most of the sequences retrieved from GenBank. For the ITS region, the different loci of the region were identified on the basis of terminal motifs of 18S, 5.8S and 28S loci catalogued [25]. Only the positions corresponding to ITS1, 5.8S, and ITS2 were kept in the alignment. The program Gblocks v0.91b [26] was then used to exclude poorly aligned positions of the ITS alignment with the following parameter settings: minimum number of sequences for a conserved position = 24 (minimum possible); minimum number of sequences for a flank position = 24 (minimum possible); maximum number of contiguous non-conserved positions = 4 bp, minimum block size = 4 bp, and gaps allowed within selected blocks in half of the sequences. Phylogenetic tree inference was performed using both Maximum Likelihood (ML) and Bayesian Inference (BI). The ML analyses were performed using RAxML-HPC2 [27] on the CIPRES Science Gateway [28], with default settings except the number of bootstrap replicates was set to 1,000 for both single-gene and combined gene analyses. Phylogenetic inference was first performed on each single-gene alignment, and, since no significantly supported conflict [with ML Bootstrap Support (BS) ≥ 70%] was detected, multiple-genes alignments and trees were built. Because ITS sequences were not alignable with reasonable confidence over the whole set of OTUs including sect. *Amanita* as outgroup, the four-gene alignment (β-tub, rpb2, ITS1+ITS2, and 5.8S) was restricted to sections *Phalloideae* and *Valideae*, the latter being used as the outgroup. BI was performed using MrBayes v.3.2.6 [29], with a mixed model partition. The best substitution model was determined for each partition of the data set separately using jModeltest v. 2 [30] on jmodeltest.org, with default parameters. When the best model could not be specified in MrBayes, the next more complex model was used. The selected models were K80 + G for β-tub, GTR + I + G for rpb2, GTR + G for ITS1 and ITS2, and 5.8S. The Bayesian analyses were conducted with 2 runs, each with five simultaneous Markov chains, and trees were summarized every 250 generation. The analyses were stopped after 1,000,000 generations, when the average standard deviation of split frequencies was 0.004999 for the two-gene analysis, and 0.002576 for the four-gene analysis. The burn-in phase (20% and 25% for the two-gene and four-gene analysis, respectively) was estimated by checking the stationarity in the plot generated by the sump command. The remaining trees were used to generate a majority-rule consensus tree and to compute corresponding posterior probabilities. Phylograms were visualized with FigTree ver. 1.3.1 [31].

### Nomenclature

The electronic version of this article in Portable Document Format (PDF) in a work with an ISSN or ISBN will represent a published work according to the International Code of Nomenclature for algae, fungi, and plants, and hence the new names contained in the electronic publication of a PLOS article are effectively published under that Code from the electronic edition alone, so there is no longer any need to provide printed copies.

In addition, new names contained in this work have been submitted to MycoBank, from where they will be made available to the Global Names Index. The unique MycoBank number can be resolved and the associated information viewed through any standard web browser by appending the MycoBank number contained in this publication to the prefix http://www.mycobank.org/MB/. The online version of this work is archived and available from the following digital repositories: PubMed Central and LOCKSS.

### Toxin analysis

Dried basidiomata of *Amanita ballerina* (OR1026), *A*. *brunneitoxicaria* (BZ2015-01), and *A*. *fuligineoides* (BZ2013-41) were analyzed for major toxins including α-amanitin and phalloidin. The basidiomata were pulverized to a fine powder and ca. 100 mg were extracted in methanol for 30 min. in an ultrasonic bath and the resulting liquid filtered through filter paper. The liquid phase of methanol was then evaporated to dryness. The crude extracts were dissolved in methanol and transferred to 4 mL glass vials, and dried under nitrogen, then weighed. Then crude extracts were again re-dissolved in methanol. Crude extracts were filtered by using SPME Strata™-X 33 u Polymeric RP cartridges (Phenomenex, Inc., Aschaffenburg, Germany) and analysed using high performance liquid chromatography (HPLC). Extracts profiles were compared to standards based on mass spectrum in the negative and positive ESI modes, as well as on their characteristic UV/V_is_ and retention time (R_t_). Standards of α-amanitin and phalloidin were obtained from Sigma–Aldrich, Germany.

## Results

### DNA sequence analyses

**Fig 1:**
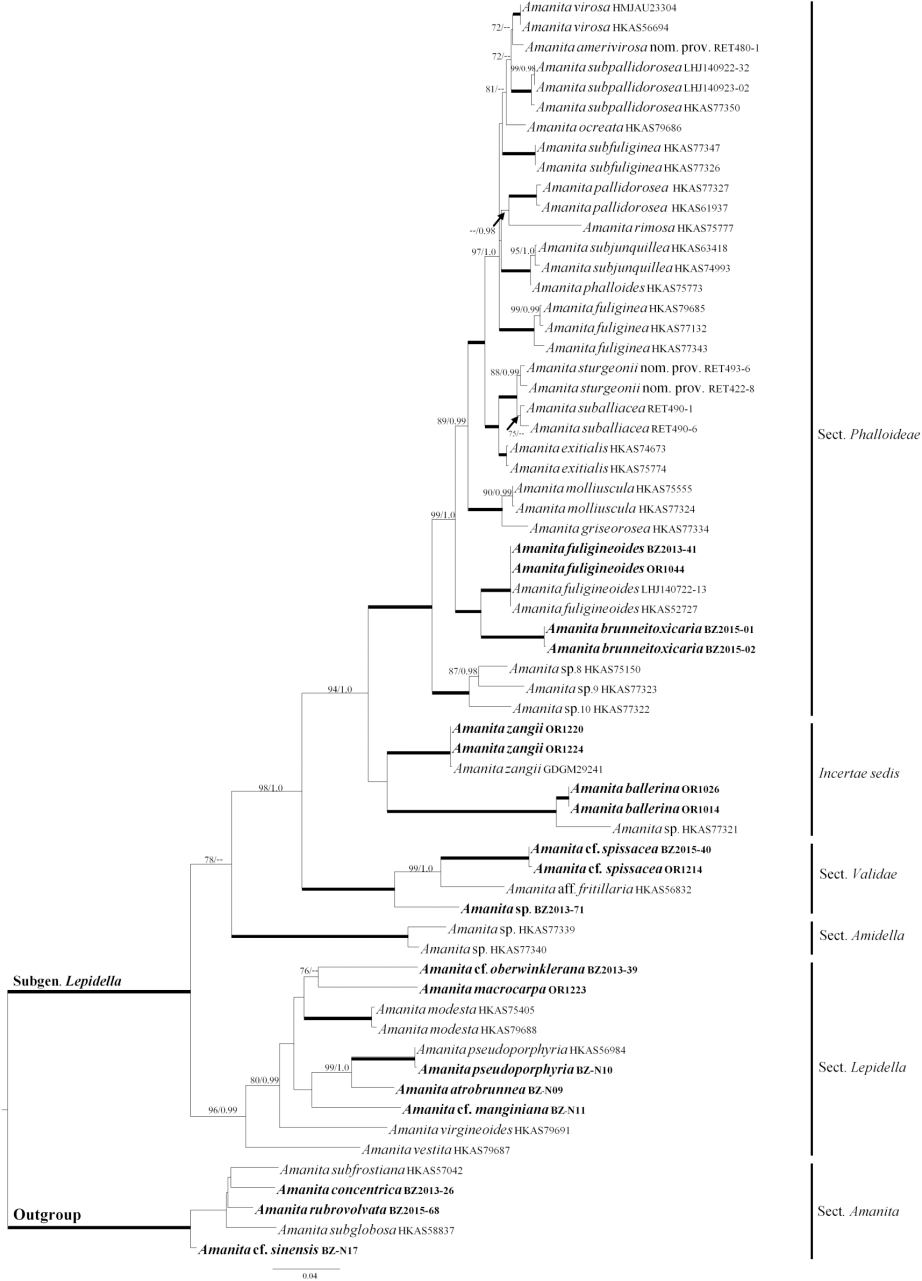
Phylogenetic tree inferred from two-gene combined dataset (β-tubulin and rpb2) using Maximum Likelihood (ML). Bootstrap values (BS) ≥70% and corresponding Posterior Probabilities (PP) ≥0.95 are shown above the branches, except when BS = 100% and PP = 1.0, which are indicated as thick branches. *Amanita* species with sequences generated in this study are highlighted in bold. Voucher collection identifiers are provided after each species name.

**Fig 2:**
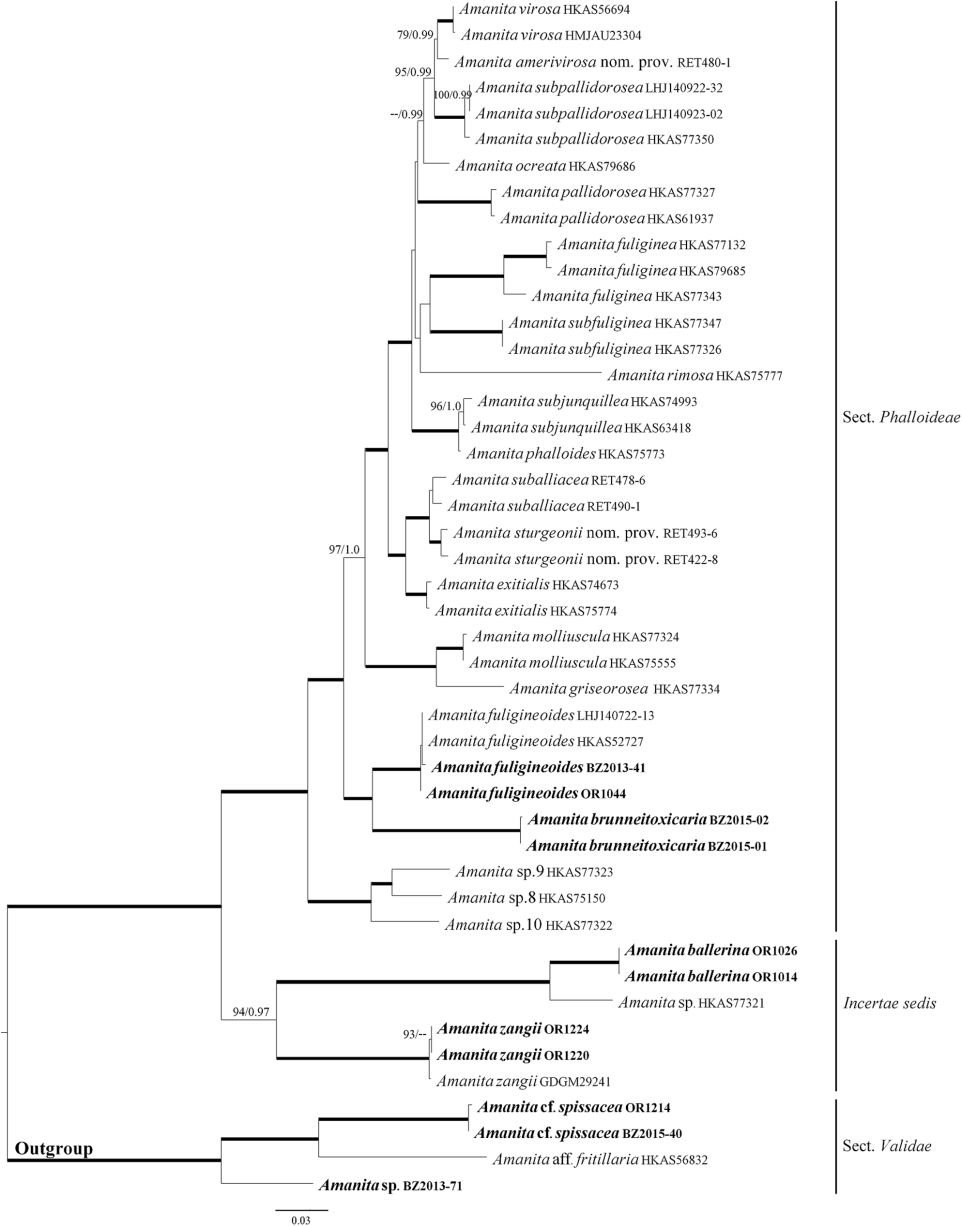
Phylogenetic tree inferred from four-gene combined dataset (β-tubulin, rpb2, ITS1+ITS2, and 5.8S) using Maximum Likelihood (ML). Bootstrap values (BS) ≥70% and corresponding Posterior Probabilities (PP) ≥0.95 are shown above the branches, except when BS = 100% and PP = 1.0, which are indicated as thick branches. *Amanita* species with sequences generated in this study are highlighted in bold. Voucher collection identifiers are provided after each species name.

In this study, each of the single-gene phylogenies showed similar tree topologies (Supporting Information S1–S3 Figs), without supported conflicts. The two-gene (β-tub and rpb2) and four-gene (β-tub, rpb2, ITS1+ITS2, and 5.8S) alignments contained 63 and 46 OTUs, and were 924 and 1,443 sites long, respectively. For the ITS1 + ITS2 alignment, Gblocks retained 375 sites (57% of a total of 653 sites). The topology of the two-gene and four-gene trees obtained in this study are consistent with previously published trees with similar taxon sampling [6, 13]. Interestingly, *Amanita ballerina* sp. nov., *A*. sp. HKAS77321, and *A*. *zangii* formed a clade that is sister to the *Phalloideae* sensu Bas [1], i.e. the death caps. This clade, however, was highly supported only in the four-gene tree (BS = 94%, PP = 0.97). In the two-gene tree BS was only 38% and the clade was not retrieved in the BI tree, where *A*. *zangii* was sister to the lethal amanitas, while *A*. *ballerina* and *A*. *sp*. HKAS77321 formed a sister clade to the poorly supported (PP = 0.73) clade comprised of *A*. *zangii* and the *Phalloideae* sensu Bas [1]. *A*. *brunneitoxicaria* sp. nov. forms a well-supported clade with *A*. *fuligineoides* (BS = 100%, PP = 1.0). Initial BLAST searches of both LSU and ITS1+5.8S+ITS2 sequences are given in **Table 2**. In this study, the tree topologies obtained from ML and BI did not show any supported conflict. Therefore, only the ML trees are shown (**Figs 1 and 2**).

**Table 2:** Results of GenBank BLAST searches for ITS and LSU sequences of *Amanita* species collected from Thailand (S = Similarity and QC = Query Cover).

Thai collection (query)	GenBank accession no.		Species	Voucher	Country	References
	ITS	LSU				
*Amanita ballerina* OR1014	KJ466416 S = 92.2%, QC = 90%	KJ466481 S = 97.3%, QC = 93%	*Amanita* sp.	HKAS77321	China	[13]
*Amanita ballerina* OR1026	KJ466416 S = 92.9%, QC = 90%	KJ466481 S = 97.3%, QC = 93%	*Amanita* sp.	HKAS77321	China	[13]
*Amanita brunneitoxicaria* BZ2015-01	KP221303 S = 88.5%, QC = 99%	KJ466485 S = 95.1%, QC = 93%	*Amanita suballiacea*	RET 490–1	USA	[13]
*Amanita brunneitoxicaria* BZ2015-02	KP221303 S = 88.5%, QC = 99%	KJ466485 S = 94.8%, QC = 91%	*Amanita suballiacea*	RET 490–1	USA	[13]
*Amanita fuligineoides* BZ2013-41	NR_119713 S = 98.9%, QC = 96%	**–**	*Amanita fuligineoides*	HKAS52316^a^	China	[12]
*Amanita fuligineoides* OR1044	NR_119713 S = 99.1%, QC = 96%	**–**	*Amanita fuligineoides*	HKAS52316^a^	China	[12]
*Amanita macrocarpa* OR1223	KC408378 S = 99.6%, QC = 96%	**–**	*Amanita macrocarpa*	GDGM31939^a^	China	[40]
*Amanita* cf. *manginiana* BZ-N11	KJ466378 S = 97.6% QC = 89%	KJ466438 S = 99.1%, QC = 97%	*Amanita manginiana* Har. & Pat.	HKAS38460	China	[43]
*Amanita* cf. *oberwinklerana*	FJ176725 S = 97.7%, QC = 96%	FJ011683 S = 99.4%, QC = 100%	*Amanita oberwinklerana* Zhu L. Yang & Yoshim. Doi	MHHNU7113	China	[12]
*Amanita pseudoporphyria* BZ-N10	KC429050 S = 99.8%, QC = 95%	KJ466450 S = 99.9%,QC = 90%	*Amanita pseudoporphyria*	HKAS56984	China	[13]
*Amanita rubrovolvata* BZ2015-68	JN943178 S = 99.1%, QC = 100%	JN941156 S = 100%, QC = 94%	*Amanita rubrovolvata*	HKAS54491	China	[38]
*Amanita* cf. *sinensis* BZ-N17	NR_119389 S = 97.6%, QC = 100%	KF02168 S = 99.7%, QC = 95%	*Amanita sinensis* Zhu L. Yang	HKAS25761^a^ KA12-1555*	China	[4], *[44]
*Amanita zangii* OR1220	KJ466432 S = 99.4%, QC = 87%	**–**	*Amanita zangii*	GDGM29241	China	[13]
*Amanita zangii* OR1224	KJ466432 S = 99.4%, QC = 87%	**–**	*Amanita zangii*	GDGM29241	China	[13]
*Amanita* cf. *spissacea* BZ2015-40	AB015683 S = 99.6%, QC = 100%	**–**	*Amanita spissacea* S. Imai	LEM960187	Japan	[41]
*Amanita* cf. *spissacea* OR1214	AB015683 S = 99.3%, QC = 100%	KU139485^b^ S = 100%, QC = 98%	*Amanita spissacea*	LEM960187 ASIS24872^a^	Japan	[41]
*Amanita* sp. BZ2013-71	AY436473 S = 95.0%, QC = 88%	**–**	*Amanita sepiacea* S. Imai	HKAS 38716	China	[43]

^a^ Holotype

^b^ Unpublished

### Taxonomy

***Amanita ballerina*** Raspé, Thongbai & K.D. Hyde, **sp. nov.** [urn:lsid:mycobank.org:names: MB 820110]. *Facesoffungi number*: FoF 03125; *Index Fungorum number*: IF552936. Type: Thailand, Chiang Mai Province, Meuang District, Doi Suthep Sub-district, Palad temple, N18°48'04"- E98°55'48", elev. 740 m, 21 July 2015, Olivier Raspé *OR1026* (holotype, SDBR-CMU OR1026; isotypes, BR 5020187254626, MFLU 16–2559).

**Fig 3:**
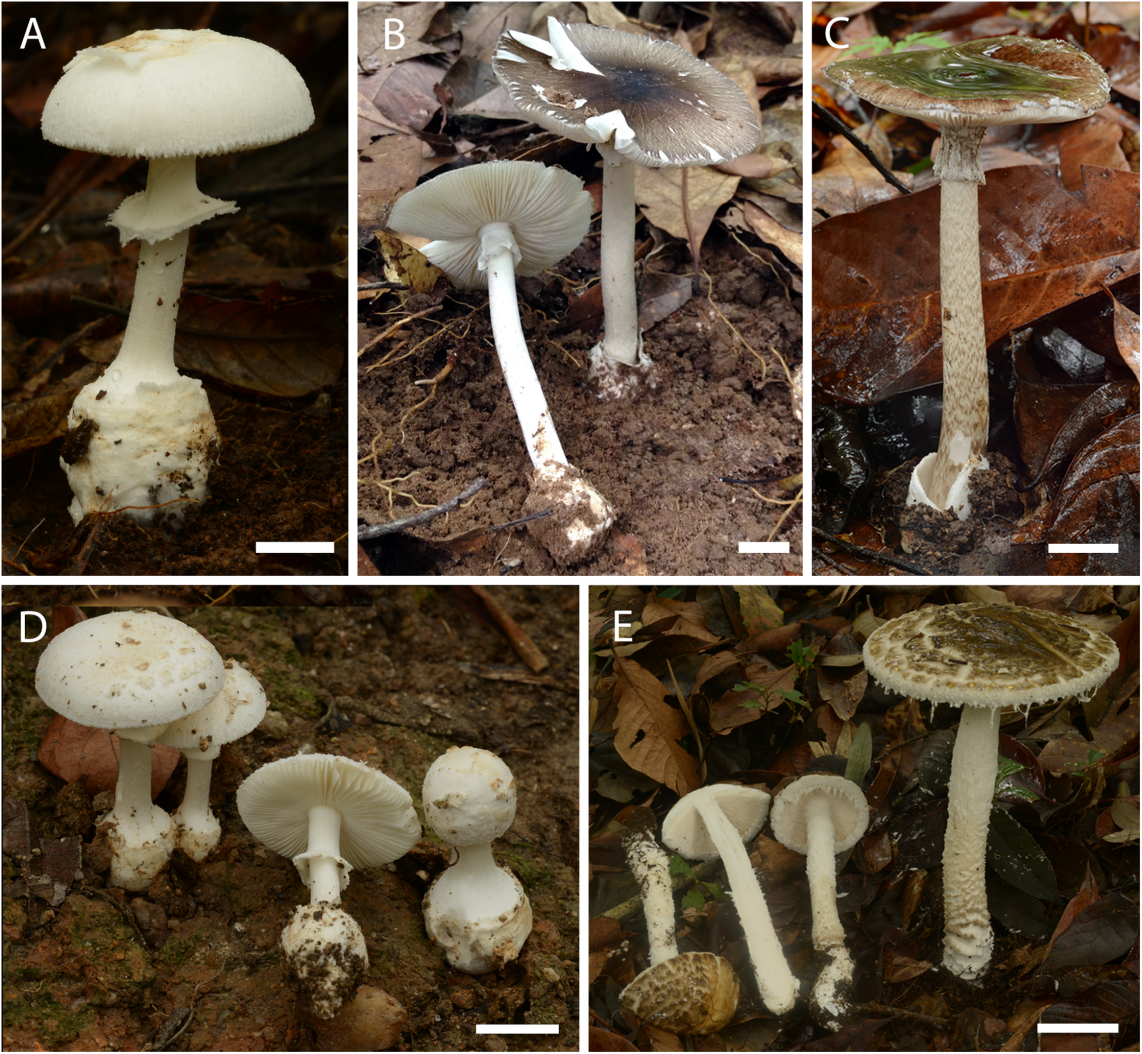
Basidiomata of the studied *Amanita* species. **(A)** *A*. *ballerina* (SDBR-CMU OR1265). (**B**) *A*. *brunneitoxicaria* (MFLU 15–3307, **holotype**). **(C)** *A*. *fuligineoides* (SDBR-CMU OR1044). **(D)** *A*. *ballerina* (SDBR-CMU OR1026, **holotype**). **(E)** *A*. *zangii* (SDBR-CMU OR1224). Scale bars: A, C = 1 cm, D-E = 2 cm.

*Etymology*:*‘****ballerina****’* refers to the cottony skirt-like partial veil, and downward tapering of the bulb, reminiscent of a ballet dancer silhouette (Fig 3).

**Basidiomata** (**Fig 3A and 3D**) small-sized. **Pileus** 34–42 mm wide, subglobose to hemispheric when young, convex to broadly convex at maturity, sometimes slightly depressed at center, dry, slightly viscid when moist, with surface under remains of general veil minutely floccose at first, becoming felted or chamois-like in age, dull white (1A2), universal veil a thin cottony layer breaking into small adherent areolate squamules or large flaky patches that are dull white to pale yellowish white (2A2); margin plane, non-striate at first then striate at maturity, non-appendiculate; context 2–3 mm thick above stem, soft to slightly hard, dull white (1A2). **Lamellae** 4–6 mm broad, narrowly sinuate, attachment to stipe, occasionally forking, close, dull white to pale yellowish white (3A2) at maturity; lamellulae of 2–3 lengths, rounded to nearly truncate. **Stipe** 43–75 × 10–26 mm (not including the bulb), nearly cylindrical, with short decurrent line at apex, bulbous, dull white, covered with fine flocculae; context stuffed, thin, yellowish white or cream (4A2). **Bulb** compressible, slightly subglobose when young, then elongate to ventricose and tapering downward, marginate up to 13 mm wide, occasionally cleft, dull white to white (2A2). **Universal veil on stipe base** a volval limb, up to 4 mm high on bulb margin, cottony-felted, white to dull white (2A2), or with patches dull white to dirty white (1A2). **Partial veil** medial, 25–35 mm below apex of stipe, persistent, cottony, skirt-like, with a ragged thickened edge, striate inside, white to dull white (1A2). **Spores** white in deposit. **Odor and taste** not recorded. **10% KOH tested** not turned to yellow in dried specimens.

**Fig 4:**
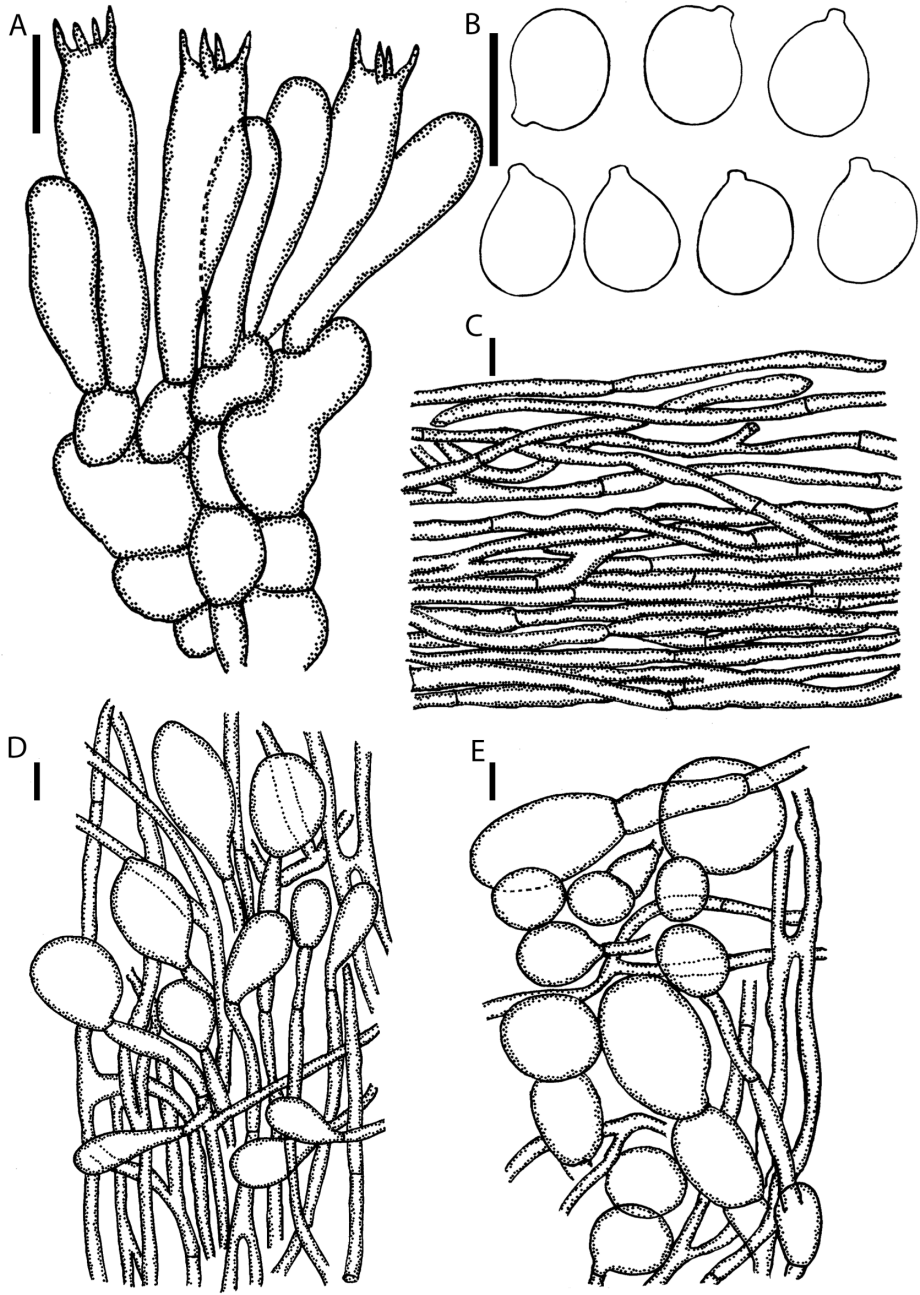
Microscopic features of *A*. *ballerina* (SDBR-CMU OR1026, holotype). **(A)** Hymenium and subhymenium. **(B)** Basidiospores. **(C)** Longitudinal section of pelius. **(D)** Longitudinal section of outer part of the partial veil. **(E)** Longitudinal section of universal veil on pileus. Scale bars: A = 20 μm, B-E = 10 μm.

**Lamellar trama** bilateral, divergent; mediostratum 60–80 μm wide, filamentous hyphae 4–8 μm wide, branching, hyaline, thin-walled; inflated cells with terminal ellipsoid to fusiform, 40–90 × 20–28 μm; vascular hyphae rare. **Subhymenium** (**Fig 4A**) 35–50 μm thick in 3–4 layers, with subglobose, ovoid or broadly ellipsoid cells dominating, 17–25 × 8–22 μm, subtended by concatenated partially inflated hyphal segments. **Basidia** (**Fig 4A**) 45–65 × 10–15 μm, long clavate, 4-spored, with sterigmata 5–7 μm long; clamps absent. **Basidiospores** (**Fig 4B**) [100/2/2] (6.6–) 7.5–8.9 (–9.1) × (5.7–) 6–7.5 (–7.7) μm, (**L'** = 8.1 μm; **W'** = 6.7 μm; Q = (1.01–) 1.06–1.36 (–1.45); **Q'** 1.22 ± 0.10), smooth, hyaline, colorless, thin-walled, amyloid, subglobose to broadly ellipsoid, rarely globose or elongate, rarely adaxially flattened; apiculus rather variable, sublateral, small or rarely large, up to 1.2 μm long, cylindric to truncate-conic; contents monoguttulate or granular. **Lamellar edge** sterile; filamentous hyphae 4–8 μm wide, branching, hyaline, colorless thin-walled; inflated cells, with subfusiform to subglobose dominating, 20–44 × 13–32 μm, hyaline, colorless, thin-walled. **Pileipellis (Fig 4C)** 90–120 μm thick, 2-layered; suprapellis up to 50–70 μm thick, filamentous hyphae, 3–10 μm wide, slightly gelatinized, often branching, hyaline, colorless, thin-walled, with terminal cells ellipsoid to clavate, 50–75 × 13–20 μm; subpellis up to 40–50 μm thick, filamentous hyphae 3–7 μm wide, undifferentiated hyphae, non-gelatinized, branching, hyaline or occasionally with intracellular yellowish brown pigment, thin-walled; vascular hyphae not observed. **Universal veil on stipe base** filamentous hyphae dominant, 3–10 μm wide, branching, thin-welled; inflated cells, with ellipsoidal, subfusiform to clavate terminus, 30–70 × 18–65 μm, colorless, thin walled; vascular hyphae rare. **Outer surface of universal veil on stipe base** abundant filamentous hyphae 5–12 mm wide, branching; mixed with inflated cells, oblong to ellipsoid or subglobose to obovoid terminus 40–90 × 20–82 μm. **Universal veil on pileus** (**Fig 4D**) filamentous hyphae 4–8 μm wide, frequently, hyaline, colorless, occasionally with intracellular brown pigment, slightly thick-walled; inflated cells with terminal subglobose to broadly ovoid cells dominating, occasionally broadly elongate, 37–68 × 34–55 μm, singly or 2–3 in chain. **Stipe trama** longitudinally acrophysalidic; filamentous undifferentiated hyphae, 3–11 μm wide, thin-walled, frequently branching; acrophysalides up to 167–278 × 26–33 μm, thin-walled; vascular hyphae not observed. **Partial veil** (**Fig 4E**) filamentous hyphae 2–5 μm wide, branching, hyaline, colorless, thin-walled, with ellipsoidal to oblong ellipsoidal terminus, sometimes subglobose, 48–71 × 17–35 μm, colorless, thin-walled; vascular hyphae not observed.

**Habitat:** solitary or scattered on the ground in evergreen Fagaceae hill forest or mixed deciduous Dipterocarpaceae/Fagaceae forest.

**Additional specimens examined:** Thailand, Chiang Mai Province, Meuang District, Doi Suthep Sub-district, Scenic Viewpoint 2, N18°47.7'-E98°55.2' elev. 950 m, 20 July 2015, Olivier Raspé *OR1014* (SDBR-CMU *OR1014*, BR 5020187248731);—, Bike trail below Phuping Palace, N18°47'50"-E98°54'21'', elev. 1170 m, 21 July 2016, Olivier Raspé *OR1265* (SDBR-CMU OR1265, BR 5020187249769).

**Known distribution:** Currently only known from Doi Suthep-Pui National Park, northern Thailand.

**Remarks:** *Amanita ballerina* is characterized by its small, white basidiomata, floccose pileus with cottony layer breaking into small adherent, dull white areolate squamules or large flaky patches, and elongate to ventricose, tapering downward bulb with short, cottony-felted volval limb. Importantly, the pileus of *A*. *ballerina* is non-striate when young but striate at maturity and sublimbate. Because the development of the volva (or egg-like) stage of the basidioma is underground,adhering soil causes the patches of the universal veil remnants to turn greyish orange (5B4-6). At first sight, *Amanita ballerina* shows some morphological similarities with the group of poisonous white or whitish taxa in sect. *Phalloideae*, such as *A*. *molliuscula*, *A*. *pallidorosea*, *A*. *parviexitialis*, *A*. *rimosa*, and *A*. *virosa*. However, *A*. *ballerina* can be easily distinguished from each of those taxa because of their globose to subglobose basal bulb, apical to subapical partial veil, and globose to subglobose basidiospores. Moreover, the floccose layer and the striations on the pileus of *A*. *ballerina* are in contradiction with the circumscription of sect. *Phalloideae* in the sense of Bas [1]. It should also be noted that *A*. *ballerina* has a floccose universal veil that leaves a friable limb on the stipe base, which no species in the *Phalloideae* has. In contrast, multiple such species are known in section *Lepidella* [1]. The bulb shape of *A*. *ballerina* and the nature of the volva are reminiscent of species of *Amanita* section *Lepidella* strips *Limbatula*, including: *A*. *limbatula* Bas, *A*. *parva* Murrill, and *A*. *praelongispora* Murrill, which have a universal veil leaving volval limb on stipe base or sublimbate stipe base [1]. *Amanita* section *Lepidella* strips *Microlepis*, e.g. *Amanita sphaerobulbosa Hongo* and *Amanita abrupta* Peck, are somewhat similar to *A*. *ballerina* in size and color, but both have small persistent pyramidal to subconical warts on the pileus and clamps at the base of basidia.

Phylogenetically, *A*. *ballerina* does not show a close relationship with either members of the *Phalloideae* with white basidiomata that contain deadly toxins or with sect. *Lepidella*. In the multigene phylogenetic tree, *Amanita ballerina*, *A*. *zangii*, and *A*. sp. HKAS77321 collected from tropical East Asia [13] form a clade sister to the *Phalloideae* sensu Bas [1] that is not closely related to sect. *Lepidella*. Unfortunately, no morphological description of *A*. sp. HKAS77321 is available for critical comparison of important morphological characters. Finally, even though nrLSU was not included in our phylogenetic analyses, blast results of *A*. *ballerina* nrLSU sequence against GenBank did not show close similarity with the available sequences from subsect. *Limbatulae* [i.e *Amanita cylindrispora* Beardslee (AY325867), *A*. *gilbertii* Beauseign (AY3458871), *A*. *mutabilis* Beardslee (HQ539714), and *A*. *praelongispora* Murrill (HQ539726)].

***Amanita brunneitoxicaria*** Thongbai, Raspé & K.D. Hyde, **sp. nov.** [urn:lsid:mycobank.org:names: MB 820111]. *Facesoffungi number*: FoF 03126; *Index Fungorum number*: IF552937. Type: Thailand, Songkhla Province, Hat Yai District, Kho Hong, N7°00'25.2"- E100°30'27.9", elev. 50 m, 16 June 2015, B. Thongbai *BZ2015-01* (holotype, MFLU 15–3307; isotype, BR 5020187250505).

*Etymology*:*‘****brunneitoxicaria****’* refers to the brown basidiomata and presence of deadly toxins.

**Fig 5:**
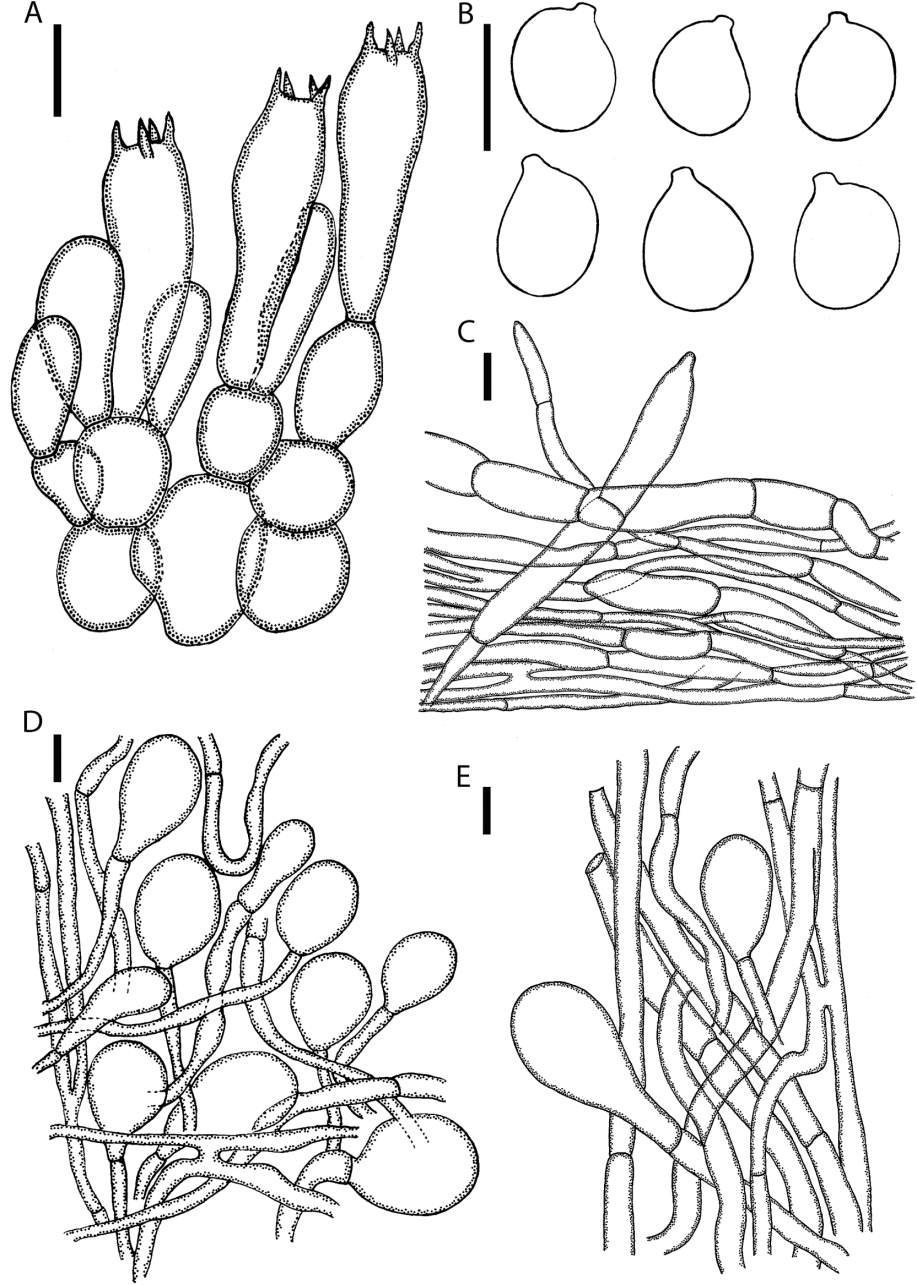
Microscopic features of *A*. *brunneitoxicaria* (holotype MFLU 15–3307). **(A)** Hymenium and subhymenium. **(B)** Basidiospores. **(C)** Longitudinal section of pelius. **(D)** Longitudinal section of outer part of the partial veil. **(E)** Longitudinal section of outer surface of universal veil on stipe base. Scale bars: A = 20 μm, B-E = 10 μm.

**Basidiomata** (**Fig 5B**) small- sized. **Pileus** 35–45 mm wide, convex to plane at maturity, dull, dry, slightly viscid when moist, virgate, greyish brown, virgae mouse grey to bronze (5E3-5 to 5-6F5-7) over white background, darkest at center (5F6 or 7F4-7), greyish brown (5E3-6E3) towards margin, universal veil remnants lacking; margin slightly downturned to plane, non-striate, non-appendiculate; context 2–4 mm thick above stem, soft to slightly hard; pale yellowish or cream (4A2-4A3). **Lamellae** 4–6 mm broad, free, close to subdistant when mature, white; lamellulae of 3–6 lengths, attenuate to nearly truncate. **Stipe** 50–60 × 10–12 mm, nearly cylindrical, flaring at apex, white to dull greyish white (1A1-1B1), with white to pale greyish brown fine flocculae; context stuffed, thin, yellowish white. **Bulb** subglobose to slightly elongated, cottony, up to 20–23 mm wide, white or dirty white (1A1, 4A2). **Universal veil on stipe base** volvate to saccate with volval limb, up to 3 mm high on bulb margin, cottony, sometimes free from stipe base, inner surface greyish, outer surface white (1A1). **Partial veil** apical, 10–13 mm below apex of stipe, membranous, frail, skirt-like, with wavy edge, white (1A1). **Spores** white in deposit. **Odor and taste** not recorded.

**Lamellar trama** bilateral, divergent; mediostratum 30–50 μm wide, filamentous hyphae abundant, 2–7 mm wide; ellipsoidal inflated cells 35–48 × 15–25 μm; vascular hyphae rare. **Subhymenium** (**Fig 5A**) 30–45 μm thick in 2–3 layers, with globose, subglobose to ellipsoidal cells dominating, 11–21 × 10–19 μm, subtended by concatenated partially inflated hyphal segments. **Basidia** (**Fig 5A**) 22–58 × 10–17 μm, clavate, 4-spored with sterigmata 4–5 μm long; clamps absent. **Basidiospores** (**Fig 5B**) [100/2/2] 7.9–9.8 (–10) × (6.6–) 6.8–7.7 (–7.8) μm, (**L'** = 8.13 μm; **W'** = 6.68 μm; Q = (1.1–) 1.2–1.42 (–1.47); **Q'** 1.27 ± 0.30), smooth, hyaline, colorless, thin-walled, amyloid, broadly ellipsoid to ellipsoid, rarely subglobose, rarely adaxially flattened; apiculus rather variable, sublateral, small or rarely large, up to 1 μm long, cylindric to truncate-conic; contents monoguttulate or rarely granular. **Lamellar edge** sterile; filamentous hyphae 3–7 μm wide, hyaline, colorless, thin-walled; inflated cells, mixed with sometimes ovoid, globose to subglobose dominating, 17–24 × 34–47 μm, colorless, thin-walled; vascular hyphae rare. **Pileipellis** (**Fig 5C**) 80–200 μm thick, 2-layered; suprapellis up to 50–120 μm thick, filamentous hyphae, 3–10 μm wide, slightly gelatinized, often branching, hyaline, colorless, thin-walled; inflated cells, with terminal cells ellipsoid to clavate, 50–150 × 20–30 μm; subpellis up to 30–80 μm thick, filamentous hyphae 3–7 μm wide, undifferentiated hyphae, non-gelatinized, branching, hyaline or often with intracellular yellowish brown pigment, thin-walled; vascular hyphae scattered. **Universal veil on stipe base** filamentous hyphae abundant 3–10 μm, branching, colorless, thin-walled; inflated cells, with subfusiform to clavate terminus, 40–78 × 32–70 μm, colorless, thin-walled, vascular hyphae rare. **Outer surface of universal veil on stipe base** (**Fig 5D**) filamentous hyphae dominant, 3–10 μm, undifferentiated hyphae, branching, colorless, thin-walled; mixed with inflated cells, with subglobose to ellipsoid terminus 130–144 × 60–79 μm. **Universal veil on pileus** not observed. **Stipe trama** longitudinally acrophysalidic; filamentous hyphae, 3–11 μm wide, thin-walled, frequently branching; acrophysalides up to 150–220 × 20–62 μm, thin-walled; vascular hyphae not observed. **Partial veil** (**Fig 5E**) filamentous hyphae 3–6 μm wide, branching, hyaline, colorless, thin-walled; inflated cells with ellipsoidal to oblong ellipsoidal or sometimes subglobose, or elongate terminus, 20–29 × 17–25 μm, colorless, thin-walled; vascular hyphae not observed.

**Habitat:** solitary on the ground in evergreen forest.

*Additional specimens examined*: Thailand, Songkhla Province, Hat Yai District, Kho Hong, N7°00'25.2"- E100°30'27.9", elev. 50 m, 16 June 2015, B. Thongbai *BZ2015-02* (MFLU 15–3308, BR 5020187251533).

**Known distribution:** Currently only known from southern Thailand.

**Remarks:** *Amanita brunneitoxicaria* is characterized by its small dark greyish brown basidioma, mouse grey to bronze virgate surface that is darkest at center, greyish brown towards pileus margin, subglobose to slightly elongated bulb with saccate volva, and the wavy edge of the apical partial veil. The dark pileus of *A*. *brunneitoxicaria* is similar to other lethal species including *A*. *fuliginea*, *A*. *fuligineoides*, *A*. *griseorosea* and *A*. *subfuliginea*, originally described from China, and *A*. *alauda* Corner & Bas, *A*. *elephas* Corner & Bas, and *A*. *privigna* Corner & Bas, originally described from Singapore.

Morphologically, *A*. *brunneitoxicaria* is similar to *A*. *fuliginea*, *A*. *fuligineoides*, and *A*. *subfuliginea* in having small to medium basidiomata with innately virgate pileus surface and 4-spored basidia. *Amanita fuliginea* is most similar but differs by its globose to subglobose basidiospores. In addition, *A*. *fuliginea* was reported to contain both α-amanitin and phalloidin [15], whereas our study of *A*. *brunneitoxicaria* revealed the presence of α-amanitin only. *A*. *fuligineoides* and *A*. *subfuliginea* have a brownish stipe covered with fibrillose squamules in a spiral or concentric pattern, while *A*. *brunneitoxicaria* has a stipe that is white to pale greyish brown, and the fine flocculae do not form a spiral or concentric pattern. *Amanita griseorosea* can be distinguished from *A*. *brunneitoxicaria* by its pinkish lamellae, the medial position of the partial veil, as well as 2-spored basidia. According to the phylogenetic analysis, most species with dark brown basidiomata do not seem to be closely related and fall into several different clades.

***Amanita fuligineoides*** P. Zhang & Zhu L. Yang [12] [urn:lsid:mycobank.org:names: MB 515097]

**Basidiomata** (**Fig 3C**) large-sized. **Pileus** 60–80 mm wide, parabolic to hemispheric when young, convex to plane or uplifted at maturity, dry, slightly viscid when moist, smooth, concentrically virgate, light brown to brown aspect, virgate fibrils grayish brown (5C-E2-4) to yellowish brown to dark brown (5D-6F6-7) over light brown (6C-D5-6) to yellowish brown (5C-D5-6) ground and at margin, fibrils coalesced and darker at center; universal veil remnants not observed; margin incurved to flaring upward, non-striate, non-appendiculate; context 2–3 mm thick above stem, soft to slightly hard; white. **Lamellae** 6–8 mm broad, free, crowded, white (1A1); lamellulae of 2–4 lengths, attenuate. **Stipe** 100–110 × 10–12 mm (length does not include bulb), nearly cylindrical or slightly tapering upwards, bulbous, white to dull white ground, covered with grayish brown (5C-E2-4), yellowish brown to dark brown (5F6-7) fibrils or squamules in concentric or spiralled zonations; context stuffed, soft, white to yellowish white (1A1-1A2). **Bulb** compressible, subclavate to napiform, slightly elongated downward, marginate, up to 15–17mm wide, white to dirty white (1A1-1A2). **Universal veil on stipe base** large volval limb on bulb margin up to 33 mm high, white. **Partial veil** subapical, 10–13 mm below apex of stipe, membranous, thin, skirt-like, persistent, inside whitish; outside pale grayish brown, slightly striate. **Spores** white in deposit. **Odor and tasted** not recorded.

**Lamellar trama** bilateral divergent; mediostratum 25–33 μm wide; filamentous hyphae 3–5 μm wide; mixed with abundant inflated elements, with clavate to fusiform terminus, 40–60×12–18 μm. **Subhymenium** 20–28 μm thick in 3–4 layers, with broadly clavate or ovoid cells dominating, 9–17 × 8–15 μm, subtended by concatenated partially inflated hyphal segments. **Basidia** 30–45 × 12–16 μm, narrowly clavate to clavate, mostly 4-, occasionally 2-spored, with sterigmata 6–7 μm long; clamps absent. **Basidiospores** [100/2/2] (7.2–) 7.6–9.2 (–10.5) × (6.6–) 7.2–9.0 (–9.5) μm, (**L'** = 8.5 μm; **W'** = 8.2 μm; Q = (1.00–) 1.01–1.12 (–1.18); **Q'** = 1.08 ± 0.04), smooth, hyaline, colorless, thin-walled, inamyloid, globose to subglobose; apiculus variable, sublateral, rather small, up to 1.3 μm long, truncate-cylindric to rarely truncate-conic; contents monoguttulate or rarely granular. **Lamellar edge** sterile; filamentous hyphae 3–8 μm wide, hyaline, colorless, thin-walled; inflated cells with mostly globose to subglobose dominating, 10–15 × 8–13 μm, colorless, occasionally single or 2–3 in chain, thin-walled; vascular hyphae rare. **Pileipellis** 70–90 μm thick, 2-layered; suprapellis up to 30–40 μm thick, filamentous hyphae, 3–6 μm wide, slightly gelatinized, branching, brown intracellular pigment, thin-walled; subpellis up to 40–50 μm thick, filamentous hyphae 3–7 μm wide, undifferentiated hyphae, non-gelatinized, branching, hyaline, colorless, thin-walled; vascular hyphae not observed. **Universal veil on the stipe base** filamentous hyphae 3–10 μm wide, interwoven, colorless, hyaline; sometimes inflated cells with ellipsoid terminus 35–60 × 14–20 μm; vascular hyphae rare. **Universal veil on pileus** not observed. **Outer surface of universal veil on stipe base** filamentous hyphae, dominantly 3–8 μm, undifferentiated hyphae, branching, colorless, thin-walled. **Stipe trama** longitudinally acrophysalidic; filamentous, undifferentiated hyphae 3–11 μm wide, thin-walled, frequently branching; acrophysalides up to 100–320 × 20–30 μm, thin-walled; vascular hyphae rare. **Partial veil** filamentous hyphae 3–6 μm wide, thick-walled, branching; abundant inflated cells with terminal single or 2–3 in chain, clavate to broadly clavate occasionally subglobose to broadly ovoid, 10–38 ×10–22 μm, colorless, hyaline, thin-walled; vascular hyphae rare.

**Habitat:** solitary on the ground in deciduous *Dipterocarpaceae* forest to hill *Fagaceae* forest.

**Specimens examined**: Thailand, Chiang Mai Province, Doi Saket District, 24 km marker on highway number 118 to Chiang Rai Province, N18°54'28''- E99°12'42'' elev. 510 m, 13 June 2013, B. Thongbai *BZ201341* (MFLU 14–0054, BR 5020187252561); Chiang Mai Province, Mae On District, Tapa Village, N19°8'11.5''- E98°45'38'' elev. 950 m, 26 July 2014, Olivier Raspé *OR1044* (SDBR-CMU OR1044, BR 5020187253599).

**Known distribution:** Subtropical regions of Central and Southern China [6] and now northern Thailand.

**Remarks:** The large-sized basidiomata of *A*. *fuligineoides* with a grayish brown to fuliginous umber pileus, persistent apical to subapical partial veil, and a subclavate to napiform blub with a firm limbate universal veil are characteristic of this species. *Amanita fuligineoides* was originally described from forests dominated by *Fagaceae* in China at elevations of 900–1,200 m. [12]. The Thai collections were found in a mixed *Dipterocarpaceae-Fagaceae* forest at elevations of 510–950 m, and agree with salient features reported in the protologue, especially with respect to the rather large basidiomata and the presence of globose to subglobose spores. The gray-brown to dark gray pileus of *A*. *fuligineoides* also occurs in *A*. *fuliginea*, *A*. *griseorosea*, and *A*. *subfuliginea* [6]. *Amanita fuliginea* was originally described from Japan but is widely distributed in China and Thailand [3, 4, 32]. *Amanita fuliginea* and *A*. *fuligineoides* are very similar in morphological features. However, *A*. *fuliginea* differs from the Thai collections by having smaller-sized basidiomata and a subglobose bulb. The molecular analysis indicates that they are not closely related. A previous study reported α-amanitin and phalloidin from both *A*. *fuliginea* and *A*. *fuligineoides* [15] which is in agreement with the toxins detected in the Thai collections of *A*. *fuligineoides*. *Amanita griseorosea* and *A*. *subfuliginea* have significantly smaller basidomata. *Amanita fuligineoides*, despite the similar morphological similarities was not closely related in the molecular analysis to either *A*. *griseorosea* or *A*. *subfuliginea*.

### Toxins

**Fig 6:**
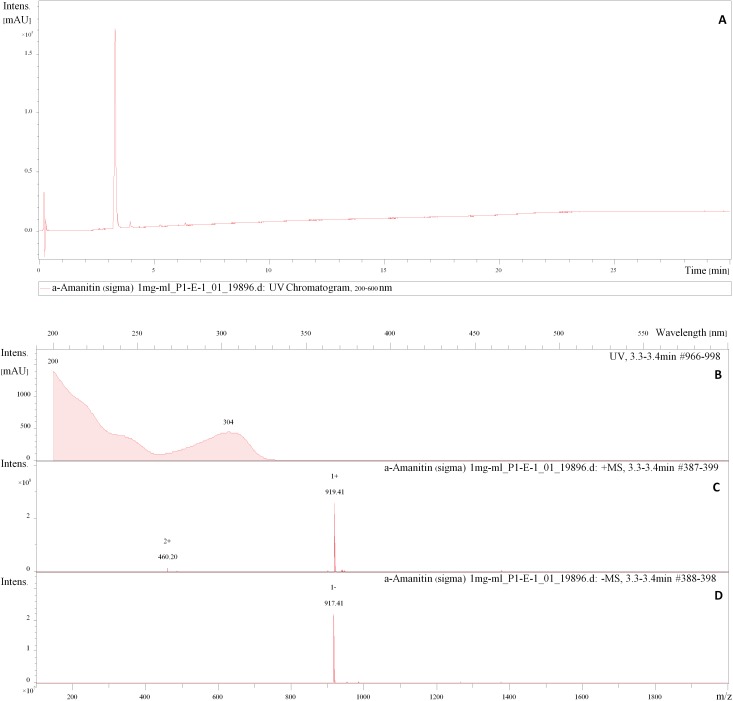
HPLC analysis of a standard of α-amanitin obtained from Sigma–Aldrich. **(A)** The peak of α-amanitin detected on the chromatogram at absorption from 200–304 nm. **(B)** Signal UV chromatogram of α-amanitin detected at 3.3–3.4 mins. **(C)** Molecular weight of 919.41 in the positive ESI mode. **(D)** Molecular weight of 917.41 in the negative ESI mode.

**Fig 7:**
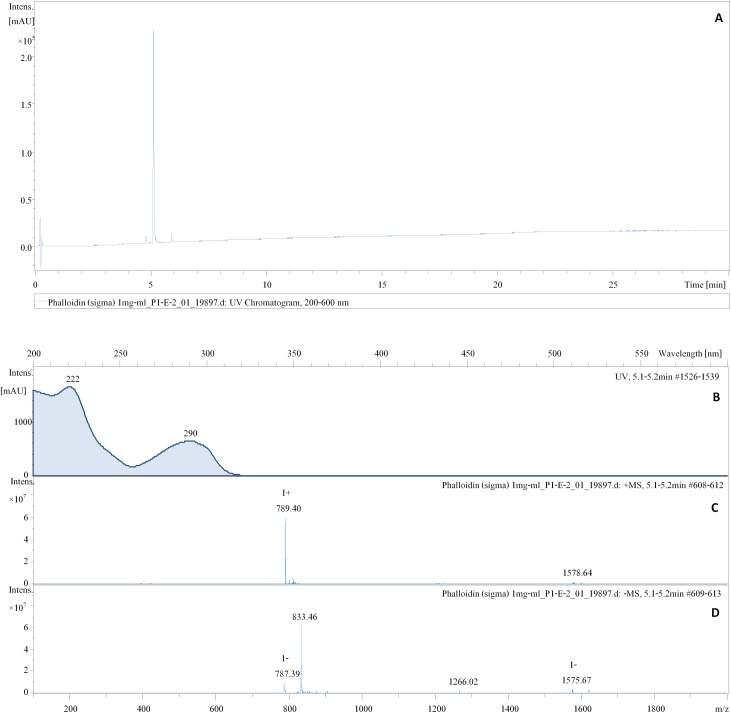
HPLC analysis of a standard of phalloidin obtained from Sigma–Aldrich. **(A)** The peak of phalloidin detected on the chromatogram at UV absorption from 222–290 nm. **(B)** Signal UV spectrum of phalloidin detected at 5.1–5.2 mins. **(C)** Molecular weight of 789.40 in the positive ESI mode. **(D)** Molecular weight of 787.39 in the negative ESI mode.

**Fig 8:**
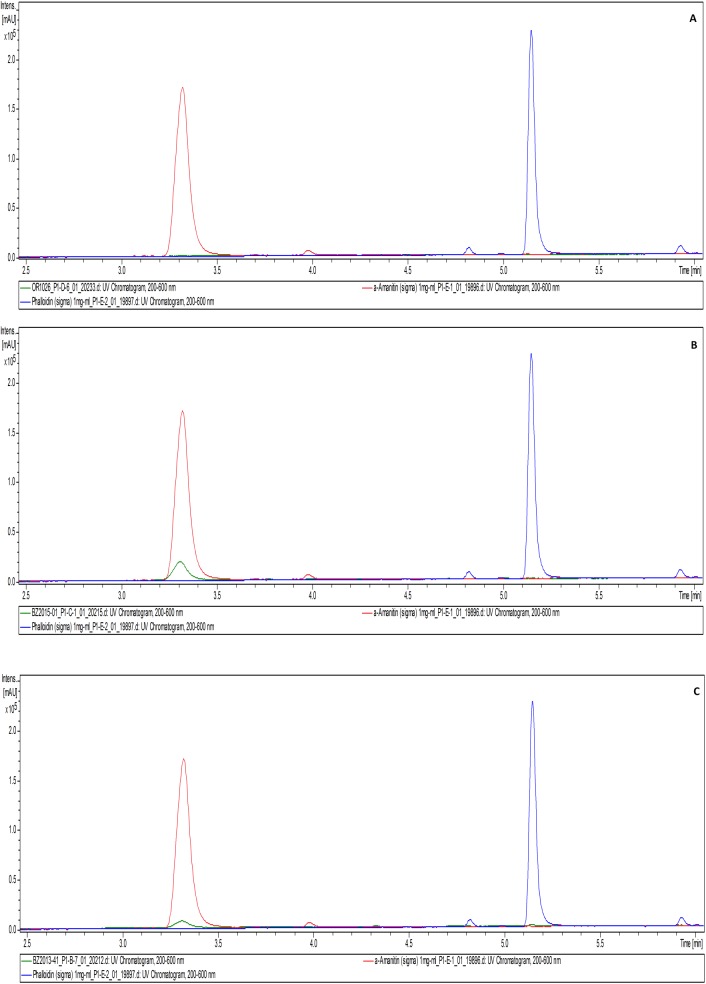
HPLC analysis of the crude extract of Thai *Amanita* collections (in green) compared with a standard of α-amanitin (in red) and phalloidin (in blue). **(A)** Chromatogram of the crude extract of *A*. *ballerina* showing non-detection of α-amanitin and phalloidin. **(B)** Chromatogram of the crude extract of *A*. *brunneitoxicaria* showing detection of α-amanitin and no detectable phalloidin. **(C)** Chromatogram of the crude extract of *A*. *fuligineoides* showing detection of both α-amanitin and phalloidin.

HPLC analysis of the α-amanitin standard showed a mass peak [M+H+H_2_O]^+^ of 919.41 amu in the positive ESI mode and [M+H-H_2_O]^-^ of 917.41 amu in the negative ESI mode, characteristic UV/Vis with an absorption maximum at 304 nm, and a retention time (R_t_) at 3.3–3.4 mins (**Fig 6**). The palloidin standard showed a mass peak [M+H+H_2_O]^+^ of 789.40 amu in the positive ESI mode, and [M+H-H_2_O]^-^ of 787.39 amu in the negative ESI mode, characteristic UV/Vis with an absorbed maximum at 290 nm, and a retention time (R_t_) at 5.1–5.2 mins (**Fig 7**). Crude extracts of Thai *Amanita* taxa were compared with these standards which showed major peaks at the R_t_ of the standards with matching mass spectra in negative ESI mode, as well as their characteristic UV/Vis. Neither α-amanitin or phalloidin were observed in *A*. *ballerina* (**Fig 8A)**. Several peaks corresponding to amatoxins were detected in the extracts of *A*. *brunneitoxicaria* (**Fig 8B)** and *A*. *fuligineoides* (**Fig 8C)**. However, quantification was only accomplished for α-amanitin and phalloidin for which standards were available. In *A*. *fuligineoides*, 6.7 mg α-amanitin per gram of dry weight and 1 mg of phalloidin per gram of dry weight were detected. *Amanita brunneitoxicaria* contained approximately 21.5 mg of α-amanitin per gram of dry weight. *A*. *brunneitoxicaria* contained only traces of α-amanitin and no phalloidin was present. Extensive evaluation of the HPLC-MS results tentatively revealed the presence of additional minor peaks that may correspond with other amatoxins. Cycloamanide C (R_t_ 5.7 min; molecular weight 886 Da; Gauhe and Wieland [33]), phallisin (R_t_ 4.3 min; molecular weight 804 Da [34]) and phalloin (R_t_ 5.9; molecular weight 772 Da [35]) were detected in *A*. *fuligineoides*, whereas *A*. *brunneitoxicaria* yielded only cycloamanide C. These results are based on the mass spectrometric data in comparison with characteristics of the amatoxins reported in the literature and would have to be confirmed by preparative isolation of the compounds, which would afford the availability of large quantities. In any case, the present study only proves the presence of the hazardous toxins in the new species *A*. *brunneitoxicaria* and additional analyses would have to be carried out on additional collections of the respective species in order to ascertain their toxicogenic potential.

## Discussion

This study, along with other recent publications [16, 36], illustrates the high diversity of *Amanita* species in Thailand, suggesting that more taxa remain to be discovered and documented. First, *Amanita ballerina* is a new species in an interesting clade that so far contained only a few species. Second, *A*. *brunneitoxicaria* clusters with *A*. *fuligineoides*, which in a previous publication was the only taxon in the lethal amanitas clade VIII [13]. Third, *Amanita brunneitoxicaria* is the first new *Amanita* species to be discovered in southern Thailand. To date, most of the taxonomic research on *Amanita* in Thailand has been focused on northern Thailand and the discovery of *A*. *brunneitoxicaria* suggests that additional study is needed in other parts of the country with different mycorrhizal hosts and ecology. Additional collections of *A*. *zangii* [16] were made during this investigation in Chiang Mai province in hill forest associated with *Castanopsis* spp. and *Quercus* spp. or *Lithocarpus* spp. at elevations of 1450 m, suggesting that *A*. *zangii* (**Fig 1E**) is not a rare species in northern Thailand.

So far, studies of *Amanita* have devoted much attention to species producing toxins, but molecular studies of *Amanita* toxin genes have been only recently conducted [8–10]. Research has begun to examine the toxins as well as phylogenies and evolutionary relationships among toxin-encoding genes [13, 15, 37, 42]. Here, we report on an initial investigation of the presence of toxins in two species of *Amanita* sect. *Phalloideae* and one species, *A*. *ballerina*, whose taxonomic placement is uncertain, all collected in Thailand. Screening for both α-amanitin and phalloidin showed that neither toxin was present in *A*. *ballerina*, as is also the case for *A*. sp. HKAS77321 and *A*. *zangii* [13]. The possibility of the presence but lack of expression of toxin-encoding genes in those species should be explored.

The position of *A*. *zangii* and of stirps Hesleri to which it is morphologically related has been previously discussed [13, 16, 23]. Previous molecular analyses showed that *A*. *zangii* and *A*. sp. HKAS77321 formed a moderately supported clade sister to a clade comprised of lethal amanitas (*Amanita* sect. *Phalloideae* sensu Bas [1]), and the former was interpreted as an early diverging lineage in the *Phalloideae* [13, 16]. In the present four-gene phylogenetic analysis, *A*. *ballerina* is a third taxon belonging to that interesting clade. It should be noted, however, that this clade was not supported in the two protein-coding gene analysis (BS = 38% only). In both the two-gene and four-gene analyses in the present study, *Amanita ballerina* and *A*. sp. HKAS77321 formed a strongly supported clade sister to *A*. *zangii*. Similarly to *A*. *zangii*, the suite of morphological characters in *A*. *ballerina* is somewhat in conflict with its placement in the *Phalloideae*. They are indeed reminiscent of *A*. *limbatula*, a rare species known from only a few collections, currently included in stirps *Limbatula* of sect. *Lepidella* [7]. A significant difference between *A*. *ballerina* and species of stirps *Limbatula*, however, is the presence of clamp connections in the latter [1]. Unfortunately, no sequence of *A*. *limbatula* is available to confirm the phylogenetic affinities of this species, and no specimen in good condition was available to us. *Amanita ballerina* shares a similar elongate to ventricose bulb morphology and pileipellis structure with *A*. *zangii*. *A*. *ballerina* has a striate margin when mature, while *A*. *zangii* has an appendiculate margin. Neither of these characters conform to the circumscription of sect. *Phalloideae* sensu Bas [1], which raises questions about placement of these two taxa. The sequence of *A*. sp. HKAS77321 also falls into the interesting clade with *A*. *ballerina* and *A*. *zangii*, but unfortunately no morphological information is available for this taxon. There is thus growing evidence that there is a divergent lineage sister to lethal amanitas, which could be interpreted as an early diverging lineage in sect. *Phalloideae*, or perhaps as a completely new section. Careful morphological and sequence analyses of members of stirps *Hesleri* and stirps *Limbatula* is required. Further exploration of *Amanita* diversity and historical biogeography in South-East Asia and Australia, which seems to be a hotspot of early diverging *Amanita* lineages. [13,14], is critical and could reveal more members of this clade, and help elucidate morphological and molecular synapomorphies to support or refute the hypothesis of a new section.

## Supporting information

S1 Fig Phylogenetic tree inferred by Maximum Likelihood analysis of β-tubulin sequences. Bootstrap values (BS) ≥70% are shown above the branches. *Amanita* species with sequences generated in this study are highlighted in bold. Voucher collection identifiers are provided after each species name.
(EMF)

S2 Fig Phylogenetic tree inferred by Maximum Likelihood analysis of rpb2 sequences. Bootstrap values (BS) ≥70% are shown above the branches. *Amanita* species with sequences generated in this study are highlighted in bold. Voucher collection identifiers are provided after each species name.
(EMF)

S3 Fig Phylogenetic tree inferred by Maximum Likelihood analysis of ITS1+5.8S+ITS2 sequences. Bootstrap values (BS) ≥70% are shown above the branches. *Amanita* species with sequences generated in this study are highlighted in bold. Voucher collection identifiers are provided after each species name.
(EMF)

## References

[1] Bas C. Morphology and subdivision of *Amanita* and a monograph of its section *Lepidella*. Persoonia. 1969; 5:285–573.

[2] Tulloss RE, Ovrebo CL, Halling RE. Studies on *Amanita* (Amanitaceae) from Andean Colombia. Mem New York Bot Gard.1992; 66:1–46.

[3] Yang ZL. Atlas of the Chinese species of Amanitaceae. Beijing: Science Press 2015 213 p.

[4] Yang ZL. Die Amanita-Arten von Südwestchina. Bibl Mycol. 1997; 170:1–240.

[5] Saccardo PA. Sylloge Fungorum. vol. 9; 1887.

[6] Cai Q, Cui YY, Yang ZL. Lethal *Amanita* species in China. Mycologia. 2016; 108(5): 993–1009. doi: 10.3852/16-008 27474516 10.3852/16-008

[7] Tulloss RE, Yang ZL. (mutable text). amanitaceae.org [cited January 2017].- Available from http://www.amanitaceae.org

[8] Helfer AG, Meyer MR, Michely JA, Maurer HH. Direct analysis of the mushroom poisons α- and β-amanitin in human urine using a novel on-line turbulent flow chromatography mode coupled to liquid chromatography-high resolution-mass spectrometry/mass spectrometry. J Chromatogr A. 2014; 1325 (2014): 92–98. doi: 10.1016/j.chroma.2013.11.054 24342531 10.1016/j.chroma.2013.11.054

[9] Nayak AP, Green BJ, Beezhold DH. Fungal hemolysins. Med Mycol. 2013; 51(1):1–16. doi: 10.3109/13693786.2012.698025 22769586 10.3109/13693786.2012.698025 PMC4663657

[10] Hallen HE, Luo H, Scott-Craig JS, Walton JD. Gene family encoding the major toxins of lethal *Amanita* mushroom. PNAS. 2007; 104(48): 19097–19101. doi: 10.1073/pnas.0707340104 18025465 10.1073/pnas.0707340104 PMC2141914

[11] Li P, Deng WQ, Li TH. The molecular diversity of toxin gene families in lethal *Amanita* mushrooms. Toxicon. 2014; 83: 59–68. doi: 10.1016/j.toxicon.2014.02.020 24613547 10.1016/j.toxicon.2014.02.020

[12] Zhang P, Tang LP, Cai Q, Xu JP. A review on the diversity, phylogeographyand population genetics of *Amanita* mushrooms. Mycology. 2015; 6(2): 86–93. doi: 10.1080/21501203.2015.1042536 10.1080/21501203.2015.1042536 PMC6106075 30151317

[13] Zhang P, Chen ZH, Xiao B, Tolgor B, Bao HY, Yang ZL. Lethal amanitas of East Asia characterized by morphological and molecular data. Fungal Divers. 2010; 42: 119–133. doi: 10.1007/s13225-010-0018-4

[14] Cai Q, Tulloss RE, Tang L, Tolgor B, Zhang P, Chen ZH, Yang ZL. Multi-locus phylogeny of lethal Amanitas: implications for species diversity and historical biogeography. ‎ BMC Evol Biol. 2014; 14(143): 1–16. doi: 10.1186/1471-2148-14-143 24950598 10.1186/1471-2148-14-143 PMC4094918

[15] Sánchez-Ramírez S, Tulloss RE, Guzmán-Davalos L, Cifuentes-Blanco J, Valenzuela R, Estrada-Torres R, et al In and out of refugia: Historical patterns of diversity and demography in the North American Caesar’s mushroom species complex. Mol. Ecol. 2015; 24(23): 5938–5956. doi: 10.1111/mec.13413 26465233 10.1111/mec.13413

[16] Thongbai B, Tulloss RE, Miller SL, Hyde KD, Chen J, Zhao R, Raspé O. A new species and four new records of *Amanita* (Amanitaceae; Basidiomycota) from Northern Thailand. Phytotaxa. 2016; 286(4): 211–231.

[17] Thiers BM. (mutable text) Index Herbariorum. 2017; Available from: http://sciweb.nybg.org/science2/IndexHerbariorum.asp, continuously updated.

[18] Tulloss RE (mutable text) Biometric variables: meanings and how to define a range. Studies in the Amanitaceae. 2017; Available from: http://www.amanitaceae.org/?HowTo's&howto=8, continuously updated.

[19] Jayasiri SC, Hyde KD, Ariyawansa HA, Bhat J, Buyck B, Cai L, et al The faces of fungi database: fungal names linked with morphology, phylogeny and human impacts. Fungal Divers. 2015; 74: 3–18. doi: 10.1007/s13225-015-0351-8

[20] Index Fungorum. 2016; Available from: www.indexfungorum.org, continuously updated.

[21] MycoBank. 2016; Available from: www.mycobank.org, continuously updated.

[22] Miller SL. New and interesting species of Russula from the southeastern United States 1. Russula billsii. Mycotaxon. 2004; 89(1): 31–38.

[23] Tulloss RE, Kuyperb TW, Vellingac EC, Yangd ZL, Hallinge RE, Gemlf J, et al The genus *Amanita* should not be split. Amanitaceae. 2016; 1(3): 1–16.

[24] Katoh K, Kuma K, Toh H, Miyata T. MAFFT version 5: improvement in accuracy of multiple sequence alignment. Nucleic Acids Res. 2005; 33: 511–518. doi: 10.1093/nar/gki198 15661851 10.1093/nar/gki198 PMC548345

[25] Rodríguez-Caycedo C, Goldman N, Tulloss RE. nrITS sublocus terminal motifs in the Amanitaceae. Studies in the Amanitaceae. 2016; Available from http://www.amanitaceae.org?nrITS+Sublocus+Termini+in+the+Amanitaceae

[26] Castresana J. Selection of conserved blocks from multiple alignments for their use in phylogenetic analysis. Mol. Biol. Evol. 2000; 17(4): 540−552. 10742046 10.1093/oxfordjournals.molbev.a026334

[27] Stamatakis A. RAxML Version 8: A tool for Phylogenetic Analysis and Post-Analysis of Large Phylogenies. Bioinformatics 10.1093/bioinformatics/btu033. 2014. Available from:http://bioinformatics.oxfordjournals.org/content/early/2014/01/21/bioinformatics.btu033.abstract. 10.1093/bioinformatics/btu033 PMC3998144 24451623

[28] Miller MA, Holder MT, Vos R, Midford PE, Liebowitz T, Chan L, et al. The CIPRES portal. 2009; Available from: http://www.phylo.org/portal2/home

[29] Ronquist F, Teslenko M, van der Mark P, Ayres D, Darling A, Höhna S, et al MrBayes 3.2: Efficient Bayesian phylogenetic inference and model choice across a large model space. Systematic Biology. 2011; 61: 539–542. 10.1093/sysbio/sys029 PMC3329765 22357727

[30] Darriba D, Taboada GL, Doallo R, Posada D. jModelTest 2: more models, new heuristics and parallel computing. Nat. Methods. 2012; 9: 772. 10.1038/nmeth.2109 PMC4594756 22847109

[31] Rambaut A. FigTree. 2009; Available from: http://tree.bio.ed.ac.uk/

[32] Sanmee R, Tulloss RE, Lumyong P, Dell B, Lumyong S. Studies on Amanita (Basidiomycetes: Amanitaceae) in Northern Thailand. Fungal Divers. 2008; 3(2): 97–123.

[33] Gauhe A, Wieland T. Über die Inhaltsstoffe des grünen Knollenblätterpilzes, LI. Die Cycloamanide, monocyclische Peptide; Isolierung und Strukturaufklärung eines cyclischen Heptapeptids (CyA B) und zweier cyclischer Oktapeptide (CyA C und CyA D). Eur J Org Chem. 1977; (5): 859–868.

[34] Wieland T, Rempel D, Gebert U, Buku A, Boehringer H. Über die Inhaltsstoffe des grünen Knollenblätterpilzes, XXXII. Chromatographische Auftrennung der Gesamtgifte und Isolierung der neuen Nebentoxine Amanin und Phallisin sowie des ungiftigen Amanullins. Eur J Org Chem. 1967; 704(1): 226–236. 10.1002/jlac.19677040124 5628714

[35] Wieland T, Mannes K. Über die Giftstoffe des grünen Knollenblätterpilzes 13. Mitteilung. Phalloin, ein weiteres Toxin. Angew Chem. 1957; 69(11): 389–389.

[36] Li GJ, Hyde KD, Zhao RL, Hongsanan S, Abdel-Aziz FA, Abdel-Wahab MA et al Fungal diversity notes 253–366: taxonomic and phylogenetic contributions to fungal taxa. Fung Divers. 2016; 78(1): 1–237. doi: 10.1007/s13225-016-0366-9

[37] Tang S, Zhou Q, He Z, Luo T, Zhang P, Yang ZL, Chen J, Chen Z. Cyclopeptide toxins of lethal amanitas: Compositions, distribution and phylogenetic implication. Toxicon. 2016; 120(2016): 78–88. 27476461 10.1016/j.toxicon.2016.07.018

[38] Cai Q, Tang LP, Yang ZL. DNA Barcoding of economically important mushrooms: A case study on lethal amanitas from China. Plant Divers Resour. 2012; 34(6): 614–622.

[39] Li HJ, Xie JW, Zhang S, Zhou YJ, Ma PB, Zhou J, Sun CY. *Amanita subpallidorosea*, a new lethal fungus from China. Mycol Prog. 2015; 14(43): 1–11. doi: 10.1007/s11557-015-1055-x

[40] Deng WQ, Li TH, Li P, Yang ZL. A new species of *Amanita* section *Lepidella* from South China. Mycol Progress. 2014; 13(2): 211−217. doi: 10.1007/s11557-013-0906-6

[41] Oda T, Tanaka C, Tsuda M. Molecular phylogeny of Japanese *Amanita* species based on nucleotide sequences of the internal transcribed spacer region of nuclear ribosomal DNA. Mycoscience.1999; 40: 57–64.

[42] Yarze JC, Tulloss RE. Acute liver injury and renal failure due to poisonous mushroom (*Amanita bisporigera*) ingestion. Am J Gastroenterol. 2012; 107(5): 790–791. doi: 10.1038/ajg.2012.29 22552246 10.1038/ajg.2012.29

[43] Zhang L, Yang J, Yang Z. Molecular phylogeny of eastern Asian species of *Amanita* (Agaricales, Basidiomycota): taxonomic and biogeographic implications. Fungal Divers. 2004; 17: 219–238.

[44] Kim CS, Jo JW, Kwag YN, Oh J, Shrestha B, Sung GH, Han SK. Four newly recorded *Amanita* species in Korea: *Amanita* sect. *Amanita* and sect. *Vaginatae*. Mycobiology. 2013; 41(3): 131–138. doi: 10.5941/MYCO.2013.41.3.131 24198667 10.5941/MYCO.2013.41.3.131 PMC3817227

